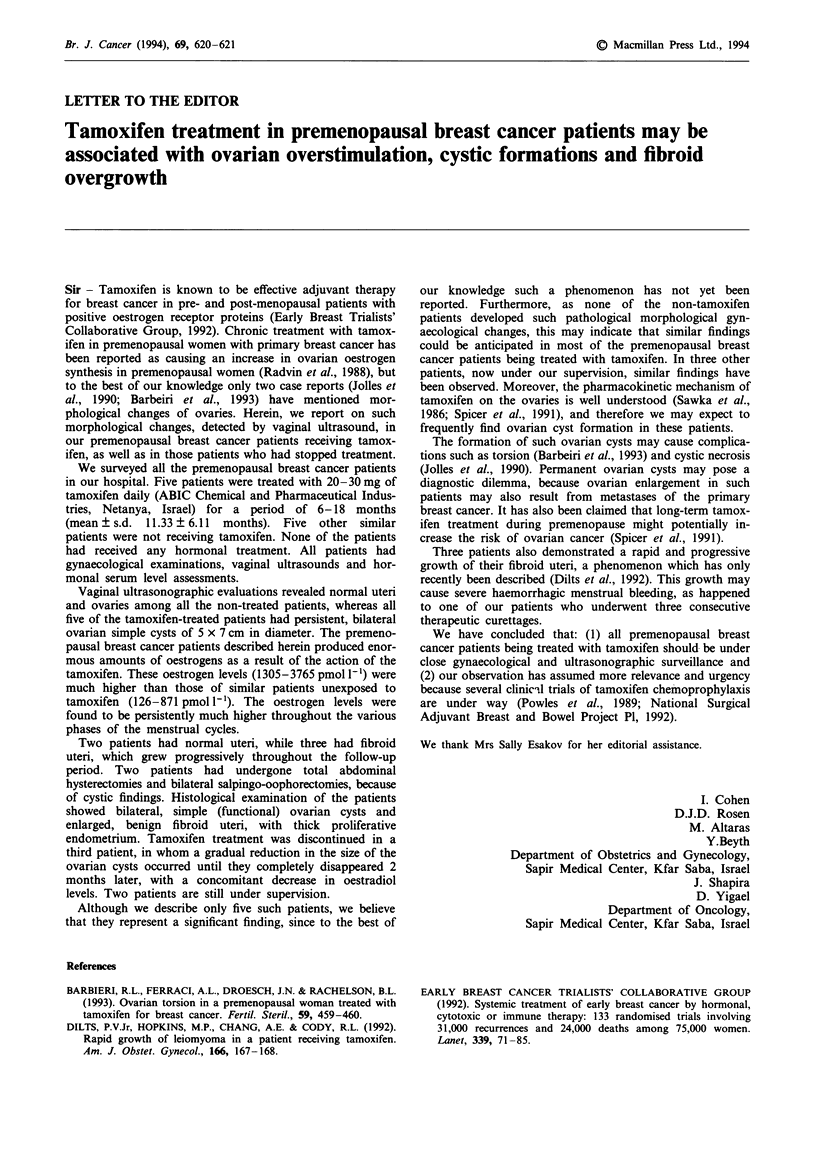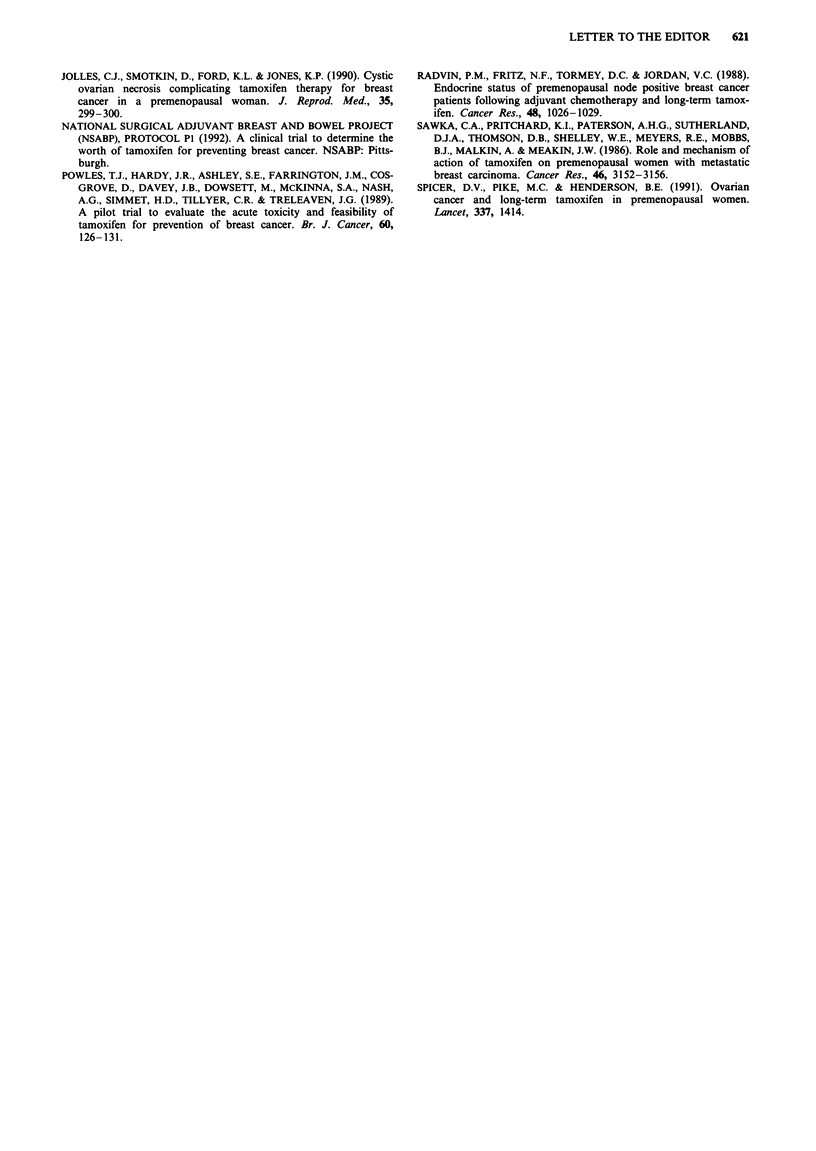# Tamoxifen treatment in premenopausal breast cancer patients may be associated with ovarian overstimulation, cystic formations and fibroid overgrowth.

**DOI:** 10.1038/bjc.1994.116

**Published:** 1994-03

**Authors:** I. Cohen, D. J. Rosen, M. Altaras, Y. Beyth, J. Shapira, D. Yigael


© Macmillan Press Ltd., 1994

LETTER TO THE EDITOR

### Tamoxifen treatment in premenopausal breast cancer patients may be associated with ovarian overstimulation, cystic formations and fibroid overgrowth

**Sir** – Tamoxifen is known to be effective adjuvant therapy for breast cancer in pre- and post-menopausal patients with positive oestrogen receptor proteins (Early Breast Trialists' Collaborative Group, 1992). Chronic treatment with tamoxifen in premenopausal women with primary breast cancer has been reported as causing an increase in ovarian oestrogen synthesis in premenopausal women (Radvin *et al.*, 1988), but to the best of our knowledge only two case reports (Jolles *et al.*, 1990; Barbeiri *et al.*, 1993) have mentioned morphological changes of ovaries. Herein, we report on such morphological changes, detected by vaginal ultrasound, in our premenopausal breast cancer patients receiving tamoxifen, as well as in those patients who had stopped treatment.

We surveyed all the premenopausal breast cancer patients in our hospital. Five patients were treated with 20–30 mg of tamoxifen daily (ABIC Chemical and Pharmaceutical Industries, Netanya, Israel) for a period of 6–18 months (mean $\pm$ s.d. $11.33 \pm 6.11$ months). Five other similar patients were not receiving tamoxifen. None of the patients had received any hormonal treatment. All patients had gynaecological examinations, vaginal ultrasounds and hormonal serum level assessments.

Vaginal ultrasonographic evaluations revealed normal uteri and ovaries among all the non-treated patients, whereas all five of the tamoxifen-treated patients had persistent, bilateral ovarian simple cysts of $5 \times 7$ cm in diameter. The premenopausal breast cancer patients described herein produced enormous amounts of oestrogens as a result of the action of the tamoxifen. These oestrogen levels (1305–3765 pmol $l^{-1}$) were much higher than those of similar patients unexposed to tamoxifen (126–871 pmol $l^{-1}$). The oestrogen levels were found to be persistently much higher throughout the various phases of the menstrual cycles.

Two patients had normal uteri, while three had fibroid uteri, which grew progressively throughout the follow-up period. Two patients had undergone total abdominal hysterectomies and bilateral salpingo-oophorectomies, because of cystic findings. Histological examination of the patients showed bilateral, simple (functional) ovarian cysts and enlarged, benign fibroid uteri, with thick proliferative endometrium. Tamoxifen treatment was discontinued in a third patient, in whom a gradual reduction in the size of the ovarian cysts occurred until they completely disappeared 2 months later, with a concomitant decrease in oestradiol levels. Two patients are still under supervision.

Although we describe only five such patients, we believe that they represent a significant finding, since to the best of our knowledge such a phenomenon has not yet been reported. Furthermore, as none of the non-tamoxifen patients developed such pathological morphological gynaecological changes, this may indicate that similar findings could be anticipated in most of the premenopausal breast cancer patients being treated with tamoxifen. In three other patients, now under our supervision, similar findings have been observed. Moreover, the pharmacokinetic mechanism of tamoxifen on the ovaries is well understood (Sawka *et al.*, 1986; Spicer *et al.*, 1991), and therefore we may expect to frequently find ovarian cyst formation in these patients.

The formation of such ovarian cysts may cause complications such as torsion (Barbeiri *et al.*, 1993) and cystic necrosis (Jolles *et al.*, 1990). Permanent ovarian cysts may pose a diagnostic dilemma, because ovarian enlargement in such patients may also result from metastases of the primary breast cancer. It has also been claimed that long-term tamoxifen treatment during premenopause might potentially increase the risk of ovarian cancer (Spicer *et al.*, 1991).

Three patients also demonstrated a rapid and progressive growth of their fibroid uteri, a phenomenon which has only recently been described (Dilts *et al.*, 1992). This growth may cause severe haemorrhagic menstrual bleeding, as happened to one of our patients who underwent three consecutive therapeutic curettages.

We have concluded that: (1) all premenopausal breast cancer patients being treated with tamoxifen should be under close gynaecological and ultrasonographic surveillance and (2) our observation has assumed more relevance and urgency because several clinical trials of tamoxifen chemoprophylaxis are under way (Powles *et al.*, 1989; National Surgical Adjuvant Breast and Bowel Project Pl, 1992).

We thank Mrs Sally Esakov for her editorial assistance.

I. Cohen
D.J.D. Rosen
M. Altaras
Y.Beyth
Department of Obstetrics and Gynecology,
Sapir Medical Center, Kfar Saba, Israel
J. Shapira
D. Yigael
Department of Oncology,
Sapir Medical Center, Kfar Saba, Israel

### References

BARBIERI, R.L., FERRACI, A.L., DROESCH, J.N. & RACHELSON, B.L. (1993). Ovarian torsion in a premenopausal woman treated with tamoxifen for breast cancer. *Fertil. Steril.*, **59**, 459–460.

DILTS, P.V.Jr, HOPKINS, M.P., CHANG, A.E. & CODY, R.L. (1992). Rapid growth of leiomyoma in a patient receiving tamoxifen. *Am. J. Obstet. Gynecol.*, **166**, 167–168.

EARLY BREAST CANCER TRIALISTS' COLLABORATIVE GROUP (1992). Systemic treatment of early breast cancer by hormonal, cytotoxic or immune therapy: 133 randomised trials involving 31,000 recurrences and 24,000 deaths among 75,000 women. *Lanet*, **339**, 71–85.

JOLLES, C.J., SMOTKIN, D., FORD, K.L. & JONES, K.P. (1990). Cystic ovarian necrosis complicating tamoxifen therapy for breast cancer in a premenopausal woman. *J. Reprod. Med.*, **35**, 299–300.

NATIONAL SURGICAL ADJUVANT BREAST AND BOWEL PROJECT (NSABP), PROTOCOL P1 (1992). A clinical trial to determine the worth of tamoxifen for preventing breast cancer. NSABP: Pittsburgh.

POWLES, T.J., HARDY, J.R., ASHLEY, S.E., FARRINGTON, J.M., COSGROVE, D., DAVEY, J.B., DOWSETT, M., MCKINNA, S.A., NASH, A.G., SIMMET, H.D., TILLYER, C.R. & TRELEAVEN, J.G. (1989). A pilot trial to evaluate the acute toxicity and feasibility of tamoxifen for prevention of breast cancer. *Br. J. Cancer*, **60**, 126–131.

RADVIN, P.M., FRITZ, N.F., TORMEY, D.C. & JORDAN, V.C. (1988). Endocrine status of premenopausal node positive breast cancer patients following adjuvant chemotherapy and long-term tamoxifen. *Cancer Res.*, **48**, 1026–1029.

SAWKA, C.A., PRITCHARD, K.I., PATERSON, A.H.G., SUTHERLAND, D.J.A., THOMSON, D.B., SHELLEY, W.E., MEYERS, R.E., MOBBS, B.J., MALKIN, A. & MEAKIN, J.W. (1986). Role and mechanism of action of tamoxifen on premenopausal women with metastatic breast carcinoma. *Cancer Res.*, **46**, 3152–3156.

SPICER, D.V., PIKE, M.C. & HENDERSON, B.E. (1991). Ovarian cancer and long-term tamoxifen in premenopausal women. *Lancet*, **337**, 1414.